# Two forms of yawning modulation in three months old infants during the Face to Face Still Face paradigm

**DOI:** 10.1371/journal.pone.0263510

**Published:** 2022-02-04

**Authors:** Damiano Menin, Tiziana Aureli, Marco Dondi

**Affiliations:** 1 Dipartimento di Studi Umanistici, Università degli Studi di Ferrara, Ferrara, Italy; 2 Dipartimento di Neuroscienze, Imaging e Scienze cliniche, Università di Chieti-Pescara G. d’Annunzio, Chieti, Italy; Universita degli Studi di Pisa, ITALY

## Abstract

The last decades have seen an increasing interest in the phenomenon of yawning and the dynamics of its modulation, yet no widespread consensus exists on its origins and potential functions. Although most scholars have focused on its potential physiological functions, e.g., related to thermoregulation, arousal modulation or cortisol levels and distress, an emerging line of research has been also investigating the social implications of yawning, including its hypothesized relationship with empathy. In order to explore the dynamics of yawning modulation in infants, we investigated whether a social perturbation–like the one induced by the Face to Face Still Face paradigm, a procedure designed to assess socio-emotional regulation in infants–affects yawning and self-touch hand movements behavior in three-months old infants. As the Still Face episode represents a source of mild distress, we hypothesized that during this phase yawns would be more frequent. Moreover, through the use of path analysis, we investigated potential dynamics of facilitation, inhibition or covariance between the frequencies of these behavioral patterns. Our results showed a sharp increase in self-touch hand movements as well as in the likelihood of yawning during the stressful phase of the procedure (still-face) compared with the two minutes of face-to-face interaction and the reunion episode. Regressions also showed a higher incidence of yawns among girls, consistently with the hypothesis that the analysis of yawning behavior might capture subtle differences in regulatory strategies of infants, possibly related to the transient sex-specific activation of the hypothalamic-pituitary-gonadal axis known as mini-puberty. The path analysis showed a greater consistency between the frequencies of self-touch hand movements during the three episodes of the procedure, compared with yawning. This finding could be a result of distinct yawning-regulating mechanisms being at play in different conditions, e.g., a modulation related to stress and one to social interaction. Taken together, these results suggest that human yawning regulation is an irreducibly complex and multifaceted phenomenon since early age. Moreover, the gender differences highlighted might suggest an early diversification in yawning modulation.

## Introduction

Yawning is a stereotyped phylogenetically and ontogenetically old behavioral pattern, unchanged throughout life and ubiquitous to vertebrates, yet no widespread consensus exists on its origins and potential functions [1, 2].

During the last decades, our understanding of the neurophysiological paths involved in yawn generation and modulation, as well as of the conditions and stimuli that can affect yawning behavior has significantly increased. This led to the identification of three neuro-physiological pathways involved in yawning regulation, namely a cholinergic, an oxytocinergic and an ACTH-mediated pathway [3, 4], as well as to the characterization of different classes of conditions affecting yawning patterns. In particular, human yawning behavior has been found to be modulated by a vast set of processes and conditions, including circadian rhythms [5, 6], hunger [7, 8], thermoregulation [2, 9, 10] emotional or social distress [11, 12], pain [13, 14], drowsiness [15] neurological conditions [16, 17], and the intake of different drugs [3, 18, 19]. Moreover, yawning can be induced by contagion in humans since at least five years of age [20], as well as in apes and other highly social species [21–24]. Several studies have hypothesized a role of empathy in the modulation of contagious yawning, based on the evidence that observers seem to be more susceptible to yawning contagion when they are observing a familiar person yawning [24–27]. However, Massen and Gallup [28] have argued that the link between contagious yawning and empathy is supported by inconclusive evidence and hindered by methodological limitations. As yawning has been proposed to be involved in vigilance regulation, an alternative interpretation for yawning contagion suggests that being sensitive to others’ yawns could enhance one’s ability to remain vigilant in potentially threatening situations [29]. Gallup and Meyers [29], in fact, have found that seeing another individual yawning makes the detection of snakes more rapid and effective, suggesting that yawn contagion might be related to a psychological adaptation for preserving group vigilance.

The synthesis of physiological and functional levels of analysis can shed new light on the dynamics of human and animal yawning, by linking each modulating factor to its neurophysiological substrate and studying on both levels (functional and physiological) specificities and relations between different classes of yawns.

However, efforts in addressing the issue concerning the origins of yawning did not have the same success, giving place to alternative theories each one presenting a particular function as the original reason for which animals started yawning. In the last years, in fact, yawning has been alternatively characterized as a mechanism to thermoregulate the brain [30] to regulate arousal [1, 31] or the production of cortisol [32], as well as an intrinsically social/communicative phenomenon [33]. Although some of these approaches (e.g. the thermoregulation theory) have received more attention and data backing than others during the last years, the theoretical discussion about the phylogenetic origins of yawning, as well as about the potential relationships between proximate and ultimate explanations, is still ongoing [34, 35].

A multifunctional account of yawning has been also proposed in recent years [36]. However, this approach still seems to assume that each species or group of species shares a common core function, from which other functions are derived or emerge.

Although the physiological functions of the distinct pathways involved in yawning modulation, as well as the complexity of their interactions, continues to elude the understanding of researchers from different fields, a multifunctional approach is consistent with the multifaceted character of yawning modulation. In particular, the cholinergic pathway has been proposed to be associated with sleep and hunger-related yawn regulation [37], while the ACTH-mediated pathway could induce stress or pain-related yawns [13, 38].

Oxytocin, on the other hand, has been proposed to be involved in the social modulation of contagious yawning [39, 40], but has also been linked to “the hidden sexuality of the yawn” [41]. This neuropeptide, known to play a key role in promoting mother-infant bonding, has in fact been suggested to have evolved sex-specific functional roles in social cognition [40]. Interestingly, recent studies [27, 42] found the frequency of contagious yawning to be higher in female than male adults, hypothesizing a link between this difference and the higher empathic capacity attributed to females [39]. However, we have to note that other studies did not find such gender difference in the rates of contagious yawning [43].

Despite the increase in yawning-related publications over the last decades, one aspect that has received little attention so far concerns yawning modulation in human fetuses, neonates, infants and children. This field of research is particularly relevant because it might allow to distinguish between ontogenetically primitive and derived functions and modulation mechanisms.

The Face-to-Face Still-Face paradigm (FFSF) [44], consisting in three episodes, during which the parent is required to interact playfully with the infant (Face-to-Face episode, FF), then to cease interaction maintaining a still face (Still-Face episode, SF) and finally to resume the face-to-face interaction (Reunion episode, RE), has proven to be particularly effective in highlighting individual differences in coping and interactive strategies of infants [45–49]. The FFSF paradigm was therefore deemed fit to examine the potential yawn modulation in infants facing a mildly stressful situation in the context of early social interaction.

The present study examined to what extent yawning frequencies in three months infants are modulated by the perturbation of social interaction introduced by the FFSF paradigm. As the SF episode represents a source of mild distress and yawning frequencies have been found to increase under stressful conditions, we hypothesized that during this phase yawns would be more frequent. This hypothesis is consistent with the idea according to which yawning is a mechanism to deal with stress and remain vigilant in potentially dangerous situations [29, 33].

Although the existing literature is not conclusive about the potential effect of gender on the overall magnitude of the still-face effect [50], various studies suggest that gender differences in the FFSF paradigm could be qualitative in nature, with boys showing more negative emotionality and girls displaying more self-comforting behaviors [51] and object orientation [52, 53] during SF. Therefore, to explore the hypothesis that yawning analysis captures subtle differences in infants’ regulatory strategies, we tested for potential gender differences in yawning occurrences during the FFSF procedure.

Moreover, in order to explore the relationship between yawning and other forms of behavioral regulation during the FFSF paradigm, the occurrences of self-touch hand movements across the three phases of the procedure were also analyzed. Self-directed behaviors, in fact, have been identified as a form of what is defined by ethologists as a displacement activity. Such behavioral patterns have been proposed as markers of social stress and increased autonomic arousal in nonhuman primates as well as in humans [54]. Hand movements, in particular, are known to be associated with increased arousal and emotional responses in infants and are frequently displayed during the FFSF paradigm [45, 55].

Finally, as both yawning and self-touch hand movements have been characterized as regulatory behaviors or displacement activities [1, 54, 55], through the use of path analysis, we investigated potential dynamics of facilitation, inhibition or covariance, between and within the two behavioral patterns across the three phases of the paradigm. The multiplicity of conditions and neural pathways associated with the modulation of yawning, leads us to hypothesize for this behavior a lesser coherence through the three phases of the procedure (as different modulatory mechanisms might be involved), compared to hand movements, as well as a stronger association of yawning frequencies with hand movements frequencies during the still-face episode, when both behaviors would be related with a stress-regulation mechanism. This might result in weaker, or even negative associations between yawning rates across the three phases of the paradigm, compared with self-touch hand movements, which are expected to show greater internal consistency.

## Methods

### Participants

The present sample was drawn from a larger longitudinal investigation focused on the affective and socio-cognitive development during infancy, conducted at the “G. D’Annunzio” University of Chieti-Pescara. A sample of mother-infant dyads (N = 89), who were video-recorded during the FFSF, were the focus of this study. Maternal age ranged between 28 and 43 years (M = 34.24, SD = 4.30). The infants were three-months old (M = 95.39 days, SD = 7.34), balanced for sex (44 females and 45 males), and were all healthy and born full-term. This study was carried out in strict accordance with the recommendations outlined by the American Psychological Association and the Italian Association of Academic Psychologists and the study was approved by the Department Ethics Review Board of Chieti-Pescara University (protocol # DNISCprot868). Written informed consent was obtained for all individual participants involved in the study and was signed by a parent. Ten infants (9 males and 1 female) cried during the SF episode. Because the paradigm was not completed, these dyads were excluded from further analysis, leaving 79 mother-infant dyads, including 36 male (46%) and 43 female (54%) infants.

### Procedure

Mothers were asked to come to the lab after the infant has been fed. In general, the experimental session started after 30 to 45 minutes since the last meal, and took place in the 9–11 a.m. time interval. The experimental setup required 15–20 minutes, during which infants remained in a state of calm wake. In case of excessive irritability, as well as if the infant showed a persistent condition of drowsiness, the dyad was excluded from the procedure. All of the dyads participated in the FFSF paradigm. Mothers were asked to play with their infants in a face-to-face interaction without using toys for two minutes (Face-to-Face episode, FF), stop playing and maintain a still face with neutral expression and no vocalizations for one minute (Still-Face episode, SF) and then resume the playful interaction for two minutes (Reunion episode, RE).

The infant (awake and alert) was placed in an infant seat facing the mother and the scene was video-recorded separately by three cameras, one focused on the infant, one on the mother and one including both members of the dyad. The three videos were subsequently edited and synchronized in a split-screen video. In order to enhance replicability, the study protocol is available on protocols.io at http://dx.doi.org/10.17504/protocols.io.bu5nny5e.

### Coding methods

Frame by frame behavioral analysis of video-recordings was performed by two independent coders expert in behavioral micro-analysis (with the secondary coder examining 34% of the video-recordings, N = 30), using ELAN, a professional software for the creation and management of complex annotations on video and audio (Max Planck Institute for Psycholinguistics, The Language Archive, Nijmegen, The Netherlands; http://tla.mpi.nl/tools/tla-tools/elan/).

#### Yawn coding

Yawns were identified holistically based on the following description from the *System for Coding Perinatal Behavior* (SCPB) [56], based on the action units (AUs) detailed in the comprehensive, anatomically based Facial Action Coding System for Infants and Young Children (Baby FACS) [57] and previous studies in the literature [5, 58]. The SCPB was employed in recent studies in order to code yawns and other behaviors in fetuses [59] and neonates [60].

Yawning (AU 94) is a stereotyped behavior characterized by a slow mouth opening with deep inspiration, followed by a brief apnea and a short expiration and mouth closing. One of the characteristic features of yawning is its timing, with a gradual acceleration followed by an abrupt deceleration of the facial actions involved. Yawning usually emerges from a relaxed face, initially involving mouth stretching widely open (AUs 25 + 27) and upper eyelids drooping (AU 43). Although the specific AUs accompanying yawns vary, at apex they may include tightly closed eyelids (AUs 6+7+43), flattened tongue shape (AU 76b), and swallowing (AU 80). During the plateau, brow knitting (AU 3), brow knotting (AU 4), nose wrinkling (AU 9), lateral lip stretching (AU 20), nostril dilatation (AU 38) and head tilting back (AU 53) may occur. In this phase, the expansion of the pharynx can quadruple its diameter, while the larynx opens up with maximal abduction of the vocal cords [5]. Yawning is often accompanied by limb stretching [58] and other bodily movements [56].

#### Self-touch hand movements coding

Self-touch hand movements were identified based on the following description from SCPB [56]:

These movements involve hands and arms and ends with the contact of hand or fingers with the head, face or mouth region. It is possible to distinguish between four different behavioral patterns, although in the analysis phase their scores can be aggregated. They are: *11A*. *Hand To Head Movements; 11B*. *Hand To Mouth Movements; 11C*. *Hand To Face Movements*; *11D*. *Finger-Sucking*.

Because no specific hypothesis was formulated for distinct sub-categories, only the general category was considered.

### Data analysis

Using Cohen’s Kappa, inter-rater reliability between the primary and secondary coder was calculated, with a satisfying level of agreement for all of the variables coded. In particular, reliability was assessed for the occurrence of yawning (Kappa = 0.93) and self-touch hand movements (Kappa = 1) by adopting a one-second threshold.

A multilevel Poisson regression at the minute-level, with self-touch hand movements occurrences as outcome, phase and participant’s sex as independent variables (fixed effects) and participant ID as random intercept, was run to investigate potential modulations (i.e., *still face effect* and *carry-over effect*) of this behavioral pattern across the FFSF procedure.

Considering the small number of observed yawns, a multilevel logistic regression, at the minute-level, was selected to account for skewed binomial distributions. This model included FFSF episode and participants’ sex as independent variables and participant ID as random intercept, and used to explore the modulation of yawning behavior across the procedure. Post-hoc analyses were run using the Tukey HSD test.

Finally, a path analysis was fitted in order to investigate the relationships between the number of yawns and of self-touch hand movements throughout the three phases of the procedure, adopting the maximum likelihood estimator. All analyses were carried out in the R statistical environment, version 4.0.2 [61], using the lmerTest [62] and the lavaan packages [63].

## Results

Twenty-one yawns were coded across 18 infants (23% of the sample). In particular, 33% of the females (n = 14) and 11% of the males (n = 4) yawned at least once. Moreover, 395 self-touch hand movements were observed, with similar frequencies per minute for males (M = 1.000, SD = .825) and females (M = .995, SD = .860).

### Regressions

Multilevel regressions revealed several effects of FFSF episode and sex on the dependent variables. In particular, the likelihood of observing at least a yawn during a minute of the procedure were increased for the still-face phase, β = 1.751, w(390) = 2.850, *p* = .004, and for females compared to males, β = 1.300, w(390) = 2.259, *p* = .024). Post-hoc analyses, carried out via Tukey HSD test, confirmed the higher likelihood of yawning during the still-face phase compared with the face-to-face episode (β = 1.751, *p* = .012) as well as compared with the reunion phase (β = 1.329, *p* = .038).

The number of self-touch hand movements was higher both during the still face phase, β = 0.685, t(390) = 5.381, *p* < .001, M = 1.494, SD = 1.526, and during the reunion phase, β = 0.277, t(390) = 2.328, *p* = .020, compared with the face to face, while no gender difference was found, β = -.0368, t(390) = 0.198, *p* = .843.

### Path analysis

**Fig 1:**
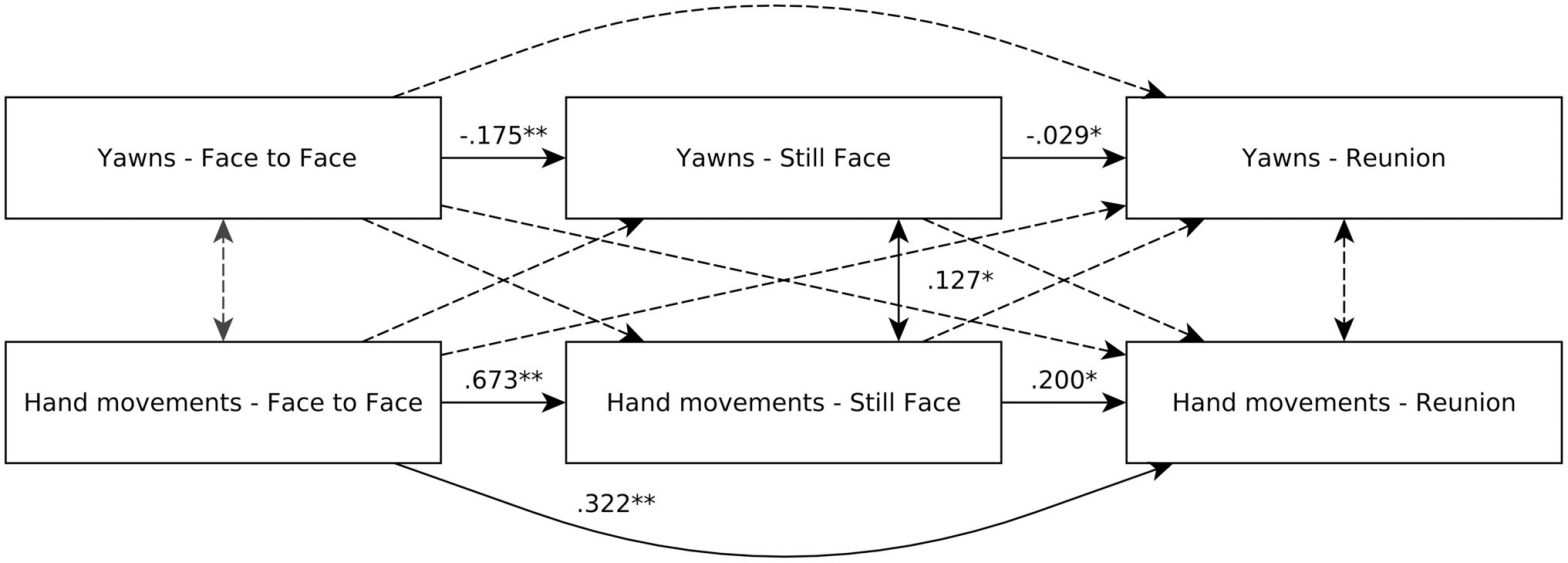
Path analysis model.

The path analysis revealed several significant effects (see Fig 1). In particular, the number of yawns per minute during the face-to-face phase showed a negative effect on the number of yawns during the still-face phase (β = -.175, *p* = .005), which in turn showed a negative effect on the number of yawns observed during the reunion phase (β = -.029, *p* = .035). Regarding self-touch hand movements, the number of events during the face-to-face phase showed a positive effect on the number of events during the still-face (β = .673, *p* = .004) and reunion phases (β = .322, *p* = .007). The number of self-touch hand movements during the still face phase also positively predicted the number of self-touch hand movements during the reunion phase (β = .200, *p* = .010). The residual covariance between the number of yawns and self-touch hand movements was significant only for the still face phase (β = .127 *p* = .023).

Unidirectional arrows indicate standardized path coefficients. Bidirectional arrows indicate covariance coefficients. Dotted lines indicate non-significant relationships (p ≥ .05). *** p < .001; ** p < .01; *p < .05.

## Discussion

In the present study, we investigated whether a social perturbation–like the one induced by the FFSF paradigm–modulates yawning and self-touch hand movements behavior in three-months old infants. Our results revealed a sharp increase in self-touch hand movements as well as in the likelihood of yawning during the still-face (SF) episode compared with the two minutes of face-to-face interaction (FF) and the reunion episode (RE).

However, since only 23% of the observed infants displayed at least one yawn, we cannot safely conclude that the FFSF procedure modulates yawning frequencies in the general population of 3-month-old infants. This state of fact could be partially due to the limited observation time characterizing the FFSF procedure, since yawning is known to be a relatively low frequency behavior in conditions of non-stimulation [64]. On the other hand, the greater incidence of yawns observed among girls is consistent with our hypothesis that the analysis of yawning behavior might capture subtle differences in regulatory strategies of infants. The gender difference we found, with girls being more likely to yawn, while, incidentally, nine out of the ten participants who cried were males, is in fact consistent with the literature that found more auto-regulatory behaviors in girls [51]. However, considering that this is the first evidence of this difference, additional studies are needed to confirm these results and to explore its potential etiology.

In term of the hormonal profiles that have been argued to be associated with various classes of yawning-modulating factors, we might hypothesize the Still-Face effect highlighted for both sexes to be ACTH-related, being associated with a condition of mild stress, while the higher incidence of yawning in females may indicate a differential oxytocinergic modulation of yawning behavior. This finding could in fact be related with the phenomenon of mini-puberty, which determines a transient sex-specific activation of the hypothalamic-pituitary-gonadal axis, known to be involved in yawn modulation [4], mainly during the first 6 postnatal months [65–68].

The higher frequency of self-touch hand movements, which were observed in 92% of the analyzed sample, allowed to reveal a *carry-over effect*, as an increased frequency of these events compared to baseline was also found during the reunion episode. This result confirms the sensitivity of this class of behavioral patterns, as an indicator of mild social distress in the context of the FFSF procedure [44, 55].

As hypothesized, the path analysis highlighted a greater internal consistency between the frequencies of self-touch hand movements during face-to-face interaction, still face and reunion, while frequencies of yawning across phases only showed negative associations, i.e. participants who yawned during a phase of the paradigm often did not yawn during the following phase. This finding could be a result of distinct yawning-regulating mechanisms being at play in different conditions (e.g., ACTH-related during the still face phase and oxytocinergic during other phases), but could also be explained by the effectiveness of yawning in regulating e.g. brain temperature or arousal levels, as further regulation would not be required.

Taken together, these results are consistent with the hypothesis that human yawning regulation is an irreducibly complex and multifaceted phenomenon since early age. Moreover, the gender differences we found might suggest an early diversification in yawning modulation, even within the same (human) species. Although our knowledge is still too limited to adopt yawning behavior as a clinical or neurobehavioral marker, the presented results are encouraging about the feasibility of disentangling distinct modulating effects affecting the frequency of this behavior.

This study presents some limitations that should be considered when interpreting its results and planning future research. First, despite posing several questions about the relationship between hormonal and behavioral factors, this study did not directly address the question regarding the possible interplay of testosterone and oxytocin in determining gender differences in yawning rates during social interactions. In order to tackle this issue, additional research involving different age-windows and physiological measures is needed to test the possible association between hormonal profiles and yawning patterns throughout infancy. This is particularly crucial as previous studies reported inconsistent findings concerning the impact of minipuberty on sex-specific behavior [68].

Further studies could also investigate potential relationships between yawning, self-touch hand movements and other behavioral patterns of interest (e.g., smiling and behavioral distress). Furthermore, administering to mothers standardized surveys would allow to investigate the relationships between the frequencies of these behavioral patterns and other constructs (e.g., parenting styles, depression).

## Supporting information

S1 File Dataset. (CSV)

S2 File R Code for data analysis. (R)
